# Eye Movement Technique to Improve Executive Function in Patients With Stroke: A Randomized Controlled Trial

**DOI:** 10.3389/fneur.2021.599850

**Published:** 2021-03-10

**Authors:** Wen He, Yazheng Ji, Xiating Wei, Fan Wang, Feng Xu, Chengyi Lu, Qianqian Ma, Kai Wang

**Affiliations:** ^1^Rehabilitation Department, Shanghai Fourth Rehabilitation Hospital, Shanghai, China; ^2^Rehabilitation Treatment Department, Shanghai Fourth Rehabilitation Hospital, Shanghai, China

**Keywords:** stroke, executive function, rehabilitation, behavioral assessment, dysexecutive syndrome

## Abstract

**Objective:** To investigate the efficacy of eye movement technique for the treatment of executive dysfunction of patients with stroke.

**Methods:** This was a prospective, single-blinded, randomized, controlled, single-center clinical trial conducted from June 2018 to December 2019 in patients with stroke. The patients were randomized 1:1 to the routine (conventional management) and eye-move group (routine management plus eye movement technique: 5-min goal management training, 5-min computer-aided working memory, and 10 min of inhibitory control training and set conversion training). The intervention lasted 6 weeks, followed by a 4-week follow-up. The primary endpoint was the Behavioral Assessment of the Dysexecutive Syndrome (BADS) score. The secondary endpoints mainly included the Montreal Cognitive Assessment (MoCA), Wisconsin Card Sorting Test (WCST), and modified Barthel Index (MBI) scores.

**Results:** Sixty-four patients were enrolled (32/group). After the 6-week intervention, the BADS and WCST scores of the eye-move group were significantly improved than those of the routine group (all *P* < 0.05), but the effects were attenuated in certain subscores after follow-up (all *P* > 0.05). The MoCA and MBI scores of the eye-move group were significantly higher, and the reaction time was significantly lower than those of the routine group at 4 weeks after the intervention (all *P* < 0.05). After follow-up, the MBI scores of the eye-move group were still higher than that of the routine group (*P* < 0.001), but there were no differences for MoCA scores and reaction time (both *P* > 0.05).

**Conclusion:** The eye movement technique could improve the executive function of patients with stroke. These results have to be confirmed.

This was a prospective, single-blinded, randomized, controlled, single-center clinical trial (ChiCTR2000036393).

**Clinical Trial Registration:** [www.chictr.org.cn], identifier [ChiCTR2000036393].

## Introduction

A stroke is an episode of acute neurological dysfunction from either ischemic infarction or a collection of blood within the brain or ventricular system with a resultant focal injury of the central nervous system (CNS) (1). Hemorrhagic strokes are typically due to intracerebral hemorrhage (ICH) or subarachnoid hemorrhage (SAH) (1). The estimated global incidence of stroke is 2–3 per 1,000 person-years (2, 3), with older patients and patients with carotid artery stenosis or atrial fibrillation having the highest risk (4, 5).

With the development of medical treatments, the mortality rate of patients with stroke decreased (6–8). Nevertheless, many patients still have sequelae, such as motor disorders, speech disorders, and cognitive dysfunction (9, 10). About 43–78% of patients will have cognitive dysfunction, including impairments of executive function (EF), thinking speed, and spatial orientation ability (11). A previous study showed that 66.6% of the patients with stroke still had EF dysfunction 2 years after stroke, mainly at the levels of choice, planning, decision-making, and control behavior disorder in daily life (12, 13), seriously affecting the quality of life of the patients and increasing the risk of dementia (13–15). At present, most of the EF training methods are occupational therapy, computer-aided cognitive training, neurocognitive psychological rehabilitation, somatosensory interaction games, acupuncture, drug treatment, hyperbaric oxygen treatment, and transcranial magnetic stimulation (16–22). Nevertheless, the evidence for efficacy is limited (16–22), and those treatments require significant investments in manpower, material resources, and financial support, and the adverse reactions of some drugs may increase physical discomfort and reduce the quality of life of the patients (16–22). Therefore, there is a need for a training method that is simple, easy to operate, more acceptable for patients, and can always maintain a high degree of compliance and enthusiasm.

The visual system is the primary sensory system for maintaining a standing posture. Visual input provides information about the surrounding environment, posture, and head movements (23, 24). Stroke patients show a variety of eye movement disorders, including reduced saccade and difficulty retaining spatial position (25), which are caused by damage to the muscles outside the eye, the cranial nerves that supply the eye muscles, or the neural pathways that control them. Eye movement disorders affect more than 70% of stroke victims. Up to 86% of symptomatic patients with stroke or non-traumatic acquired brain injury have eye movement disorders (26). In the general population of stroke victims, 7–55% have eye movement disorders at various stages of recovery (27). Visual field and visual accuracy can affect balance and motor ability, and people with impaired visual systems often have balance problems (28). Stroke patients with eye movement disorders have difficulty maintaining normal eye positions and moving their eyes appropriately (e.g., scanning, tracking, and staring), resulting in a loss of depth perception, reduced hand-eye coordination, and significant difficulty in approaching tasks and reading (27, 29). A reduced ability to scan the visual environment may affect visual memory, recognition, and the ability to make plans and decisions (30), affecting EF. Therefore, improvement of visual attention is very important for the rehabilitation of stroke patients.

The eye movement technique is considered to be an effective method to improve postural control of stroke patients through visual feedback to regulate movement patterns in exercise training. The neurologic basis of eye training is to participate in the regulation of the vestibular reflex (VOR), which plays an important role in the perception of spatial position and balance of the human body (31). The generation and control of eye movements involve the cortex, basal ganglia, cerebellum, and brainstem, and signals from this complex network eventually converge on the eye motor neurons in the brainstem (32). During eye training, visual, auditory, vestibular, and proprioceptive receptors, as well as visuospatial perception, stimulate the central nervous system (CNS), enabling it to respond quickly and accurately to environmental changes (33). Visual and proprioceptive sensory information affecting balance control can improve agility and dynamic balance control in older adults (34). As a new way of human-computer interaction, the eye movement technique has higher efficiency and is closer to the natural behavior of humans compared with currently commonly used human-computer interaction devices such as a keyboard, mouse, and touch screen (35).

At present, studies on computer-based cognitive rehabilitation training have made some progress in China and abroad, showing effectiveness in improving cognitive functions such as memory and EF (36–38). Nevertheless, this technique is still seldom used in patients with stroke, and additional data are needed. The eye movement intervention strategy developed based on eye movement technology in this study focuses on the training of saccade, tracking, and gaze, alleviates eye movement disorders, activates the neural network of the brain, and promotes the improvement of EF and attention. The eye movement used in this study uses a restorative strategy, including those where there is direct training of the impaired function or repetitive stimulation of eye movement. In the past, it has been thought that restitutive approaches would have a limited effect on visual rehabilitation. However, treatments of convergent fusion and stereopsis through repetition training of the deficient function have been reported as effective (39). But few studies have reported the efficacy of the combination of routine rehabilitation and eye movement technique on executive function in patients with stroke.

Therefore, this study investigated an EF rehabilitation strategy based on routine rehabilitation combined with the eye movement technique. This study should provide data on its effect of EF in patients with stroke and provide a reference for further discussion on EF rehabilitation treatment strategies.

## Methods

### Study Design

This was a prospective, single-blinded, randomized, controlled, single-center clinical trial (ChiCTR2000036393).

### Ethics

This study was approved by the ethics committee of the Forth Shanghai Rehabilitation Hospital (SP2018002). All participants were informed of the study and signed the informed consent form prior to any study procedure. This study was performed according to the Declaration of Helsinki and Good Clinical Practices.

### Participants

The subjects were patients with stroke and cognitive dysfunction hospitalized in the rehabilitation ward of Shanghai Fourth Rehabilitation Hospital from June 2018 to December 2019. The inclusion criteria were: (1) diagnosis of stroke (6–8, 20, 22); (2) the first stroke ever; (3) disease course ≤ 6 months; (4) no brain atrophy or leukoaraiosis with a moderate degree or above detected by imaging examination; (5) no visual field defect or visual space neglect; (6) right-handed; (7) Montreal Cognitive Assessment (MoCA) scale <26 points, which is defined as cognitive function impairment (if the education is <12 years, 1 point is added); (8) 50–75 years of age; (9) no consciousness disorder; and (10) no history of mental illness. The exclusion criteria were: (1) severe visual and hearing impairment, color blindness, color weakness, or aphasia; (2) patients with severe heart, liver, or kidney dysfunction, respiratory failure, malignant tumor, or other serious physical diseases; (3) drug abuse or alcohol dependence; (4) taking antidepressants or other psychoactive drugs; or (5) any other factors that could affect assessment or treatment.

### Sample Size

According to the results of a preliminary study, the sample size was estimated according to the primary outcome of the Behavioral Assessment of the Dysexecutive Syndrome (BADS) as the indicator. With α = 0.05 and β = 0.1 (power = 0.9), a sample size of *n* = 25 was required, calculated using PASS 11.0. Considering a loss to follow-up of <20%, the total sample size of the two groups was estimated as 64.

### Randomization and Blinding

The patients were randomized to the routine group and eye-move group using an MS Excel-generated randomization code, in a 1:1 ratio, 32 participants per group. The allocation was performed using sequential opaque envelopes. The envelopes were opened in turn. The outcome evaluators were blind to grouping.

### Intervention

The participants in the routine group received regular cognitive rehabilitation training (40), while the participants in the eye-move group received cognitive rehabilitation based on the eye movement technique. The study lasted 10 weeks. The first 6 weeks were the intervention period, and weeks 7–10 were the follow-up period.

The interventions in the two groups are detailed in Table 1. Four parts of the intervention were common to the two groups, while the last part was different. The rehabilitation intervention in the two groups was performed 6 days a week for 6 consecutive weeks. All patients were required to finish physical therapy, occupational therapy, and swallowing and speech function training, each of which lasted 20 min. Then, the patients were required to complete their group-specific intervention, i.e., routine cognitive training for the routine group and cognitive rehabilitation strategy module training for the eye-move group, each of which lasted 20 min. Hence, the training took 80 min and was required to be completed within 1 day, but it could be completed in multiple sessions, depending on the patient's physical condition.

**Table 1:** Interventions in the two groups.

**Routine group**	**Eye-move group**
Physical therapy: Good position placement of the limb, conversion and transfer of body position, muscle strength and endurance training, range of motion exercises, balance, and coordination ability training, and gait training (20 min).	
Occupational therapy: daily life activity training, including dressing, eating, brushing teeth, washing face, and auxiliary equipment training (20 min).	
Swallowing and speech function training: oral and facial muscle training, eating training, articulating training, listening, speaking, and reading and writing training (20 min).	
Drug intervention: for neurotrophy, circulation improvement, and control of risk factors of cerebrovascular disease.	
Routine cognitive training (20 min): Card sorting, picture recognition, picture visual scanning, word retelling, short reading, picture memory, word pairing, simple calculation, discrimination, and classification.	Cognitive rehabilitation strategy module based on the eye movement technique (JZ-RZ-1020, Hangzhou Jizhi Medical Technology Co., Ltd.) (20 min): Eye movement visual training (5 min): tracking and staring training. Before training, the equipment was adjusted, and the patient took the upright position. (i) Track ball training: the patient was required to stare at the ball moving on the interface until the eye movement track points appeared at the end of the training. (ii) Skiing training: after entering the training interface, a skier slid from left to right, and the patient was instructed to keep a close eye on the skier to the end of the track. The training ended with the appearance of an eye the tracking points. (iii) Fruit cutting training: after the patient entered the training interface, fruits fell from the screen at a uniform speed from top to bottom. The patient was instructed to watch each falling fruit one by one with his eyes. After hearing the click, the fruits were cut on the interface, and the patient was instructed to quickly find the next target. The training ended with the appearance of the eye-tracking points. (iv) Commodity selection: three rows of lockers with four commodities in each row, a total of 12 kinds of medicines or books, were displayed on the interface. The patients were instructed to find the corresponding commodities one by one according to the instructions and keep staring for a certain time after finding each item until they chose all the commodities. (v) Electronic organ training: the patient was instructed to stare at either black or white keys with his eyes, and after staring at them for a certain time (difficulty level from 0.2 to 0.5 s), the system played the piano and made a sound. Computer-aided working memory (short-term memory) training (5-min): (i) Visual memory training (2–3 min): the exercise of cooking, feeding fish, archery, and other tasks were performed with the aid of vivid pictures from the computer. (ii) Auditory memory training (2–3 min): the exercise of listening to words, music listening, and recognition. Inhibitory control training and set conversion training (10 min): Inhibition control training and set switching training: by changing the color of the card (yellow, red, blue, green), changing the shape of the card (pentacle, triangle, circle, cross) and adjusting the number of graphics on the card (1, 2, 3, 4). The above training follows the principle from easy to difficult and gradually deepens the training content.

A total of four investigators guided the rehabilitation training of the patients, including two primary and two intermediate rehabilitation specialists, who were all engaged in the field of neurological rehabilitation with a working experience of 5–10 years. Before the experiment, they all received unified training and guided the patients to carry out training in a unified language. The participants were assessed face-to-face by the same rehabilitation assessment therapist (blind to grouping) before and after the intervention as well as the end of the follow-up.

The termination criteria were: (1) intolerable participants during the experiment; (2) participants were unwilling to continue the clinical trials and actively proposed to suspend the trial, and the reasons would be recorded; (3) participants failed to be assessed on time.

### Assessment and Data Collection

Depression and anxiety of enrolled patients using the Self-Rating Depression Scale (SDS) (41) and the Self-rating Anxiety Scale (SAS) (42) were recorded at baseline.

The following scales were used as evaluation tools to assess the ability of patients before and after the intervention as well as after the follow-up.

**Figure 1:**
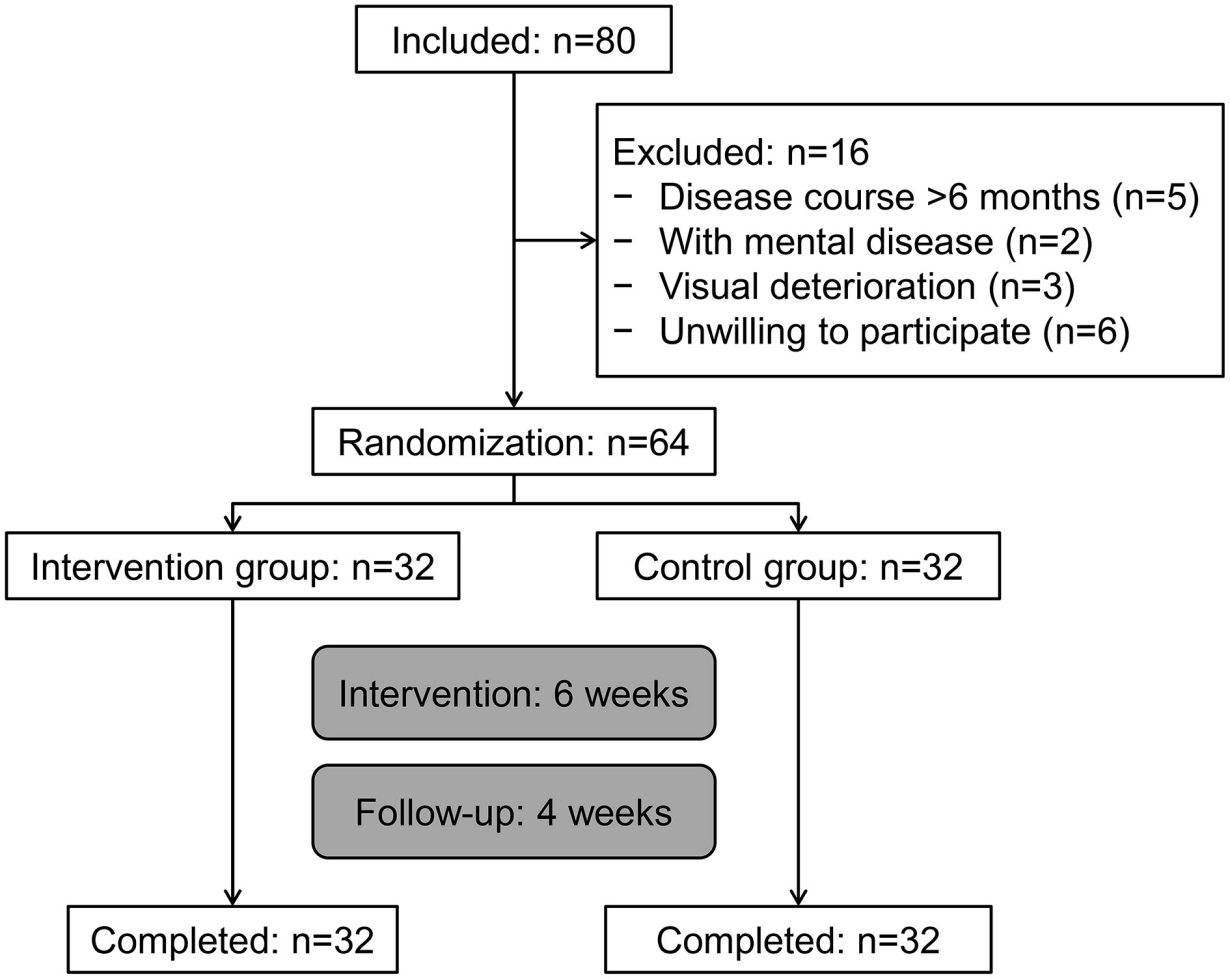
Participant flowchart.

(1) MoCA (43): The MoCA assesses visual-spatial and EF, naming, memory, attention, language, abstraction, delayed recall, and orientation. The highest score is 30 points. The higher the score, the better the cognitive function of the subject. A score of <26 points indicates cognitive dysfunction.

(2) BADS (44): the BADS is the closest method to the scene of daily life for executive function assessment. It has the characteristics of short test time, easy cooperation, and comprehensive items. The test indexes are (1) rule conversion card test (RSCT), (2) action planning test (APT), (3) key finding test (KST), (4) time judgment test (TJT), (5) zoological map test (ZMT), and the revised six-element test (MSET). The BADS assesses a patient's rule switching, planning, problem-solving, and organizational and behavioral supervision. Each test is converted to a standard score through a preliminary score (the higher the error rate, the lower the score). The single standard scores range 0–4 points. The total standard score ranges of 0–24 points, and the lower the score, the worse the executive function.

(3) Wisconsin Card Sorting Test (WCST) (45): The WCST is a traditional neuropsychological test and is a common tool to detect executive function. In this study, the computer version of the Jizhi rehabilitation diagnosis system was adopted. The test classification card has four stimulus cards and 128 classification cards, which are compiled according to color (yellow, red, blue, green), shape (pentacle, triangle, circle, cross), and number of figures (1, 2, 3, 4). The subjects are asked to classify the 128 cards according to four stimulus cards within 5 min. The subjects are not informed of the classification principle but only know whether the result of each test is true or false. The indexes in the WCST are as follows. (1) Number of classifications completed: number of classifications completed after the survey. Its value ranges from (0 to 6), indicating cognitive function, and is used to measure the extent to which subjects master the concepts of classification into different categories. (2) The correct number of responses: in the process of measurement, the correct number of responses, that is, in line with the required response principles of all responses. (3) The number of wrong responses: in the process of measurement, the number of wrong responses, that is, all responses that do not conform to the required response principles. A normal value of ≤ 45 indicates the cognitive transfer ability of the subjects. (4) Persistent error number: it means that after the classification principle is changed, the subjects cannot give up the old classification principle and stubbornly continue to classify according to the original classification principle. It reflects the problems of concept formation, the use of corrections, and the plasticity of concepts. It suggests damage to the frontal lobe function. (5) Non-persistent errors: the difference between the total number of errors and the number of persistent errors. The normal value is ≤ 24. High scores suggest inattention or confusion of thinking. (6) learning to mastering: only three or more categories can be completed to calculate, that is, the average of the difference between the percentage of false responses in the adjacent two classification stages. The normal value is ≥-10. A low score indicates that previous experience cannot be effectively applied, suggesting certain obstacles in learning ability.

(4) Reaction time test: The reaction time test system in the rehabilitation evaluation module of the Jizhi rehabilitation diagnosis system was used for assessment.

(5) The assessment of ADL ability: the modified Barthel Index (MBI) (46) was used to evaluate the ability of daily life activities of patients; a score of 100 indicates normal daily life ability.

### Endpoints

The primary endpoint was the BADS score after the intervention. The secondary endpoints were BADS scores after the follow-up, as well as the MoCA, WCST, reaction time, and MBI scores both after the intervention and at the end of follow-up.

### Adverse Events

Respiration (times /min), pulse (times /min), and blood pressure (mmHg) were used as routine safety indexes. Any adverse reactions or other discomforts to the participants during the trial were recorded, including the name of the adverse event, beginning and end time, degree, interventions taken, and recovery conditions.

### Statistical Analysis

Statistical analysis was performed using SPSS 22.0 (IBM Corp., Armonk, NY, USA). Categorical variables were presented as n (%) and were analyzed using the chi-square test or Fisher's exact test. Continuous variables were presented as means ± standard deviations (SD). The continuous variables were tested using the Shapiro–Wilk test to verify whether they met the normal distribution and using the homogeneity of variance test. If both conditions were met, the data were presented as means ± standard deviations (SD), and the independent samples *t*-test was used for inter-group comparison. Repeated measurement variance analysis was performed for comparison at different time points within each group, with the LSD *post-hoc* test. If the distribution was not normal or variance was not homogeneous, the data were expressed as median (range), and Mann–Whitney *U*-test was used for inter-group comparison, and the Friedman's rank-sum test was used for intra-group comparison. Two-tailed (except for the chi-square test) *P* < 0.05 were considered statistically significant.

## Results

### Baseline Characteristics

There were 80 patients with stroke screened. Among them, 16 cases were not in accordance with the inclusion criteria, and 6 cases were unwilling to participate in the trial. At last, a total of 64 patients were eligible and randomly assigned to the routine group and eye-move group (1:1), and all participants completed the trial, as shown in Figure 1. The demographic and clinical characteristics of the patients are shown in Table 2. There were no differences in years of education and depression and anxiety scores (all *P* > 0.05).

**Table 2:** Baseline characteristics of the participants.

**Characteristics**		**Routine group (*n* = 32)**	**Eye-move group (*n* = 32)**	***P***
Age, years, *n* (%)		67.0 ± 5.9	66.0 ± 6.9	0.628
	50–59	7 (21.9)	7 (21.9)	0.696
	60–69	10 (31.2)	13 (40.6)	
	70–75	15 (46.9)	12 (37.5)	
Sex, *n* (%)	Male	18 (56.3)	17 (53.1)	0.802
	Female	14 (43.7)	15 (46.9)	
Education, years		11.6 ± 1.8	11.7 ± 2.3	0.114
Occupation, *n* (%)	Mainly mental work	15 (46.9)	16 (50.0)	0.802
	Mainly physical labor	17 (53.1)	16 (50.0)	
Course of disease, *n* (%)	2 months	10 (31.2)	10 (31.2)	0.832
	3–4 months	13 (40.6)	11 (34.4)	
	5–6 months	9 (28.2)	11 (34.4)	
Disease nature, *n* (%)	Hemorrhage	19 (59.4)	17 (53.1)	0.614
	Infarction	13 (40.6)	15 (46.9)	
Hemiplegic side, *n* (%)	Left	16 (50.0)	18 (56.3)	0.616
	Right	16 (50.0)	14 (43.7)	
Disease location, *n* (%)	Simple basal ganglia	18 (56.3)	20 (62.5)	0.611
	Other	14 (43.7)	12 (37.5)	
SDS		58.7 ± 1.4	59.3 ± 1.3	0.084
SAS		52.8 ± 1.4	53.4 ± 1.2	0.055

### Comparison of the BADS Scores Between the Two Groups

As presented in Table 3, there were no significant differences in BADS total and subscores between the two groups before the intervention (*P* > 0.05). The BADS total scores of the eye-move group were significantly higher than those of the routine group after the intervention (median, 12 vs. 8, *P* < 0.001). However, after follow-up, the BADS scores of the eye-move group were only significantly different in the rule shift card test (RSCT) and temporal judgment test (TJT) subscores compared with those of the routine group (*P* < 0.05). In the routine group, the BADS total score improved after the intervention (median, 8 vs. 7, *P* < 0.05), but back to before after follow-up (*P* > 0.05). Similarly, there was a significant improvement in all subscores and total score of the eye-move group after the intervention (all *P* < 0.05), but there were no significant differences before treatment and after follow-up (all *P* > 0.05).

**Table 3:** Comparison of the BADS scores in each dimension between the two groups.

**Items, median (range)**	**Routine group (*****n*** **=** **32)**			**Eye-move group (*****n*** **=** **32)**			**P_**group**_**	**P_**time**_**	**P_**interaction**_**
	**Before intervention**	**After intervention**	**After follow-up**	**Before intervention**	**After intervention**	**After follow-up**			
RSCT	1 (1, 2)	1.5 (1, 3)	1 (1, 2)^e^	1 (1, 2)	2 (2, 3)^ad^	1.5 (1, 2)^a,c^	0.041	<0.001	<0.001
APT	1 (0, 2)	1 (1, 3)	1 (1, 2)	1 (0, 2)	2 (1, 3)^a,d^	1 (1, 2)^c^	0.225	<0.001	<0.001
KST	1 (0, 2)	1 (0, 3)	1 (0, 2)	1 (1, 2)	2 (1, 3)^ab^	1 (1, 3)^e^	0.004	<0.001	0.013
TJT	1 (1, 3)	1.5 (1, 3)	1 (1, 3)	1 (1, 3)	2 (1, 4)^a,d^	1 (1, 3)^a,c^	0.741	<0.001	0.013
ZMT	1 (0, 2)	1 (0, 3)	1 (0, 2)	1 (0, 2)	2 (0, 3)^a,b^	1 (0, 2)^c^	0.304	<0.001	0.004
MSET	1 (0, 2)	1 (0, 2)	1 (0, 2)	1 (0, 2)	2 (0, 3)^a,b^	1 (0, 2)^c^	0.038	<0.001	<0.001
Total	7 (4, 11)	8 (5, 15)^d^	7 (5, 10)^c^	7 (4, 11)	12 (9, 17)^f,d^	7 (5, 11)^c^	0.008	<0.001	<0.001

*Compared with the routine group in the same period: ^a^P < 0.05; ^f^P < 0.001; compared with before intervention: ^b^P < 0.05; ^d^P < 0.001; compared with after intervention: ^c^P < 0.001; ^e^P < 0.05; LSD post-hoc test was used*.

*RSCT, rule shift card test; APT, action program test; KST, key search test; TJT, temporal judgment test; ZMT, zoo map test; MSET, modified six-element test*.

### Comparison of the WCST Scores Between the Two Groups

As shown in Table 4, there were no significant differences in WCST scores in each dimension between the two groups before intervention (all *P* > 0.05). After the intervention, subscores in dimensions of completed classification number (median, 4 vs. 4, *P* < 0.05) and correct response number (median, 68 vs. 55, *P* < 0.001) in the eye-move group were significantly higher than those of the routine group. And the wrong response number (median, 42 vs. 49, *P* < 0.05), persistent error number (11 vs. 18, *P* < 0.001), non-persistent error number (median, 23 vs. 26, *P* < 0.05) were much lower in the eye-move group than the routine group. Nonetheless, after follow-up, the effectiveness of eye movement technique treatment only existed in the eye-move group regarding the correct response number (median, 60 vs. 51, *P* < 0.001), wrong response number (median, 45 vs. 51, *P* < 0.05), and persistent error number (median, 12 vs. 18, *P* < 0.05), compared with the routine group. In the routine group, there were significant differences in all WCST subscores except for the completed classification number in each dimension before and after the intervention (all *P* < 0.05). After follow-up, there were no significant differences in the correct response number (*P* > 0.05), but there were still significant differences in wrong response number, persistent response number, non-persistent response number, and time score from learning to mastering before the intervention and after follow-up (*P* < 0.05). In the eye-move group, the WCST subscores in each dimension after intervention were significantly different from those before the intervention and after follow-up (all *P* < 0.05), except for the completed classification number between before intervention and after follow-up (*P* > 0.05; Table 4).

**Table 4:** Comparison of the WCST scores in each dimension.

**Items, median (range)**	**Routine group (*****n*** **=** **32)**			**Eye-move group (*****n*** **=** **32)**			**P_**group**_**	**P_**time**_**	**P_**interaction**_**
	**Before intervention**	**After intervention**	**Follow-up**	**Before intervention**	**After intervention**	**Follow-up**			
Completed classification number	3 (2, 4)	4 (3, 4)	3 (2, 4)	3 (3, 4)	4 (3, 6)^a,b^	3 (2, 4)^e^	0.058	<0.001	0.031
Correct response number	51 (28, 63)	55 (29, 70)^d^	51 (28, 68)^c^	56 (30, 68)	68 (48, 80)^d,f^	60 (46, 75)^b,c,f^	<0.001	<0.001	<0.001
Wrong response number	53 (25, 63)	49 (20, 60)^d^	51 (22, 60)^d,e^	51 (31, 80)	42 (23, 60)^a,d^	45 (25, 70)^a,d,e^	0.243	<0.001	<0.001
Persistent error number	19 (10, 33)	18 (8, 28)^d^	18 (7, 30)^d^	18 (11, 31)	11 (8, 19)^d,f^	12 (8, 28)^a,d^	0.007	<0.001	0.003
Non-persistent error number	38 (22, 48)	26 (12, 36)^d^	27 (13, 46)^d^	36 (28, 42)	23 (11, 32)^a,d^	28 (12, 46)^b,c^	0.112	<0.001	0.022
Time from learning to mastering	−15 (−29, −10)	−11 (−24, −8)^d^	−12 (−26, −5)^b,e^	−17 (−28, −9)	−10 (−15, −1)^d^	−12 (−16, −4)^c,d^	0.381	<0.001	<0.001

*Compared with the routine group in the same period: ^a^P < 0.05; ^f^P < 0.001; compared with before intervention: ^b^P < 0.05; ^d^P < 0.001; compared with after intervention: ^c^P < 0.001; ^e^P < 0.05; LSD post-hoc test was used*.

### Comparison of the MoCA, MBI, and Reaction Time Scores Between the Two Groups

The MoCA (17.3 ± 2.3 vs. 16.0 ± 2.3, *P* = 0.03), and MBI scores (median, 65 vs. 61, *P* < 0.001) were significantly higher, and the reaction time (0.56 ± 0.07 vs. 0.60 ± 0.06, *P* = 0.022) was significantly lower in the eye-move group than those of the routine group after the intervention. However, after follow-up, there were no significant differences in MoCA scores (*P* = 0.482) and reaction time (*P* = 0.416) between the two groups, but MBI scores of the eye-move group were still higher than the routine group (median, 63 vs. 60, *P* < 0.001). Moreover, there were no significant differences in the MoCA scores of both groups before the intervention and after follow-up (both *P* > 0.05), but there were significant differences after the intervention (*P* < 0.001). The MBI and reaction time scores were all significantly different before the intervention, after the intervention, and after follow-up (*P* < 0.05; Table 5).

**Table 5:** Comparison of the MoCA scores, MBI scores, and reaction time scores.

**Items, median (range)**	**Routine group (*****n*** **=** **32)**			**Eye-move group (*****n*** **=** **32)**			**P_**group**_**	**P_**time**_**	**P_**interaction**_**
	**Before intervention**	**After intervention**	**Follow-up**	**Before intervention**	**After intervention**	**Follow-up**			
MoCA scores	14.5 ± 2.4	16.0 ± 2.3^a^	14.5 ± 2.3	14.6 ± 2.6	17.3 ± 2.3^a^	14.9 ± 2.3^b^	0.279	<0.001	<0.001
MBI scores	42 (30, 52)	61 (46, 65)^a^	60 (43, 65)^a,b^	42 (30, 55)	65 (60, 78)^a^	63 (58, 73)^a,b^	<0.001	<0.001	<0.001
Reaction time scores	0.65 ± 0.06	0.60 ± 0.06^a^	0.63 ± 0.06^b,c^	0.67 ± 0.06	0.56 ± 0.07^a^	0.62 ± 0.05^a,b^	0.577	<0.001	<0.001

*Compared with before intervention: ^a^P < 0.001, ^c^P < 0.05; Compared with after intervention: ^b^P < 0.05; LSD post-hoc test was used*.

### Adverse Events

No adverse events were observed during this trial.

## Discussion

Executive dysfunction is a feature of many patients who survived a stroke and greatly affects their quality of life (12, 13). The available rehabilitation technique proposed so far have limited efficacy for EF rehabilitation (16–22). This study investigated the effect of the eye movement technique on the executive dysfunction of patients with stroke. The results suggest that the eye movement technique could improve the executive function of patients with stroke.

There are multiple cognitive impairments in patients after stroke, and various areas also affect each other. First, the form of the breakthrough game was adopted to improve the interest, flexibility, and compliance of patients to participate in training, and then improve their emotion. Second, the selection of appropriate rehabilitation interventions and enriched treatment environments are conducive to the reorganization of brain structure and function to a certain extent. Indeed, studies showed that colorful environmental stimulation and repeated intervention training could affect nerve cells, increase the dendrites, form new neural pathways, and promote the development of brain plasticity (37, 38, 47, 48). Moreover, the eye movement technique is the first module of the rehabilitation strategy intervention and firstly focuses on the training of attention and spatial orientation. Patients with stroke often have decreased attention (49). If the patient's alertness is declined, they cannot effectively filter out the irrelevant stimulation in the procedure of information processing, which directly affects the follow-up processing (49). The intervention proposed here requires the patient's eyeball to follow the movement of the target. With the change of the target, the movement speed, scanning track, width, and direction of the eyeball change accordingly, which can activate other areas that are involved in the neural network controlling the eyeball movement, including the frontal and parietal lobes, which is conducive to the reorganization of the brain neural functions.

The results of this study showed that, compared with before the intervention and after 6 weeks of rehabilitation intervention, the scores of the BADS dimensions and WSCT dimensions (except “number of categories completed”) were significantly improved in the eye-move group. In addition, the BADS scores of the intervention group were significantly higher than those of the routine group, and other sub-scores of the WCST in all dimensions were better than those of the routine group except “learning to mastering.” These results suggest that cognitive rehabilitation training based on eye movement techniques could effectively improve the EF of stroke patients. In the cognitive rehabilitation training based on eye movement technology, the eye movement part includes tracking and staring training. The track ball and skiing training are eye movement visual tracking, while fruit cutting training, commodity purchasing training, and electronic organ training are eye movement visual staring. It is different from conventional cognitive rehabilitation training and visual training that includes only visual tracking components. In the process of eye movement gaze, patients can change the direction of the gaze voluntarily and quickly through eye movement, which is consistent with the flexibility and inhibitory control ability involved in EF. This may be one of the reasons why eye movement technology can effectively improve the executive function of stroke patients. We also analyzed the index of “learning to mastering” of the WCST, which reflects the learning ability during the implementation process, but showed no significant difference after the intervention. The possible reason is that the participants were all elderly, and their learning ability to successfully apply past experience was generally decreased.

The results of this study showed that, after the intervention, the response time of the two groups was significantly shortened and the MOCA score was significantly improved, and the improvement was more obvious in the eye-move group than in the routine group. These results suggest that rehabilitation training based on eye movement technology can effectively improve the attention and cognitive function of the patients. Direct training of attention usually requires the patients to complete a series of repetitive exercises or exercises that enable them to respond to visual or auditory stimuli. We designed the eye movement rehabilitation on the basis of cognitive rehabilitation training modules, including auditory memory like listening to the words and listening to music for identification practice, all of which required to quickly respond to the patients through word and music recognition, which at the same time also could improve the patients' EF through auditory information extraction and auditory working memory ability.

This study also showed that the “rule switching card” in the BADS and the “correct response number,” “false response number,” and “persistent error number” in the WSCT were significantly improved after the intervention compared with the routine group. This suggests that the improvement of these indicators is through the improvement of inhibitory control ability in EF, which plays a certain positive role in the selection, allocation, and persistence of attention. Eye movement technology might also help patients obtain more information, improve their response time, and improve their alertness. The improvement in attention selection, distribution, and persistence in the process of attention represent the improvement of inhibition and control ability, which further contributes to the rehabilitation of executive function.

After 4 weeks of follow-up, the BADS, WSCT, and MOCA scores and response time of patients in both groups were decreased compared with those after the intervention. This suggests that although the intervention lasted 6 weeks and achieved some results immediately after the intervention, the training time might not be enough to stabilize these changes. Hence, the intervention time might need to be extended in the future, and the training intensity might need to be increased as well. Further studies are needed. EF is one of the most complex cognitive processes, and its recovery cannot be accomplished overnight and requires a period of accumulation and persistence (50, 51). EF is a most complex cognitive process, the basis of human cognitive, emotional, and social skills, and a group of skills to independently complete purposeful and self-control behaviors (52). After a stroke, the patients need to carry out motor learning again, but this process requires individual selection, integration, organization, understanding, reasoning, and goals, and this process cannot be accomplished overnight but need time to be sustainable (18, 53).

EF also emphasizes the importance of the interactions with the environment, requires the body to eliminate or suppress irrelevant information interference, selects necessary information input, extracts, compares, and integrates relevant information from long-term memory, and regulates daily activities or acquired activities. It is this regulatory role that makes daily activities and behaviors become coordinated, orderly, and purposeful (52). Therefore, EF is closely related to adaptive behavior in daily life (52). A previous study showed that the ADL of patients depends on cognitive function to some extent, and cognitive dysfunction could cause the decline of the self-care ability of the patients (54). In the multiple regression analysis conducted by Li et al. (55), cognitive dysfunction was still an independent negative influence factor of ADL. Executive dysfunction could cause thinking rigidity, poor learning ability, low self-efficacy, and significantly reduced environmental adaptability in patients (13). Moreover, with natural aging, the EF decreases significantly, while after stroke, the EF decreases even more rapidly and more significantly (56).

This study has limitations. It was a single-center trial that might be biased by the local practice at the study hospital. There was no healthy control group. The patients were not tested for IQ, and questionnaires like the General Health Questionnaire were not used. Nevertheless, the aim of this study was not to determine the effect of eye movement training in the general population, but specifically in patients with stroke. Single treatment duration was used, and it is unknown whether the treatment effect might be consolidated using a longer treatment. The immediate and sustained effect of the intervention need to be further observed, and the neural plasticity basis of the treatment also needs to be further explored using electrophysiology, imaging, and other techniques.

In conclusion, this study indicates that rehabilitation training using the eye movement technique was beneficial to the recovery of EF and the improvement of quality of life in patients with stroke.

## Data Availability Statement

The original contributions presented in the study are included in the article/supplementary material, further inquiries can be directed to the corresponding author/s.

## Ethics Statement

This study was approved by the ethics committee of the Forth Shanghai Rehabilitation Hospital (SP2018002). The patients/participants provided their written informed consent to participate in this study. Written informed consent was obtained from the individual(s) for the publication of any potentially identifiable images or data included in this article.

## Author Contributions

WH and KW: conceptualization. CL, YJ, and XW: data curation. CL: formal analysis. YJ and XW: investigation. QM and FX: methodology and resources. FW and KW: project administration. FW: supervision. WH: roles/writing—original draft. WH and FW: writing—review & editing. All Authors read and approved the manuscript.

## Conflict of Interest

The authors declare that the research was conducted in the absence of any commercial or financial relationships that could be construed as a potential conflict of interest.
